# Reconfiguration of nucleosome-depleted regions at distal regulatory elements accompanies DNA methylation of enhancers and insulators in cancer

**DOI:** 10.1101/gr.163485.113

**Published:** 2014-09

**Authors:** Phillippa C. Taberlay, Aaron L. Statham, Theresa K. Kelly, Susan J. Clark, Peter A. Jones

**Affiliations:** 1 Epigenetics Research, Cancer Program, Garvan Institute of Medical Research, Darlinghurst, New South Wales 2010, Australia;; 2 Departments of Biochemistry and Urology, Norris Comprehensive Cancer Center, University of Southern California, Los Angeles, California 90033, USA;; 3 St. Vincent’s Clinical School, Faculty of Medicine, University of New South Wales, Darlinghurst, New South Wales 2010, Australia;; 4 Active Motif, Inc., Carlsbad, California 92008, USA;; 5 Van Andel Research Institute, Grand Rapids, Michigan 49503, USA

## Abstract

It is well established that cancer-associated epigenetic repression occurs concomitant with CpG island hypermethylation and loss of nucleosomes at promoters, but the role of nucleosome occupancy and epigenetic reprogramming at distal regulatory elements in cancer is still poorly understood. Here, we evaluate the scope of global epigenetic alterations at enhancers and insulator elements in prostate and breast cancer cells using simultaneous genome-wide mapping of DNA methylation and nucleosome occupancy (NOMe-seq). We find that the genomic location of nucleosome-depleted regions (NDRs) is mostly cell type specific and preferentially found at enhancers in normal cells. In cancer cells, however, we observe a global reconfiguration of NDRs at distal regulatory elements coupled with a substantial reorganization of the cancer methylome. Aberrant acquisition of nucleosomes at enhancer-associated NDRs is associated with hypermethylation and epigenetic silencing marks, and conversely, loss of nucleosomes with demethylation and epigenetic activation. Remarkably, we show that nucleosomes remain strongly organized and phased at many facultative distal regulatory elements, even in the absence of a NDR as an anchor. Finally, we find that key transcription factor (TF) binding sites also show extensive peripheral nucleosome phasing, suggesting the potential for TFs to organize NDRs genome-wide and contribute to deregulation of cancer epigenomes. Together, our findings suggest that “decommissioning” of NDRs and TFs at distal regulatory elements in cancer cells is accompanied by DNA hypermethylation susceptibility of enhancers and insulator elements, which in turn may contribute to an altered genome-wide architecture and epigenetic deregulation in malignancy.

Epigenetic mechanisms are necessary for normal cellular functions and are mitotically heritable through many cellular divisions. Disruption of faithful maintenance of epigenetic processes, including DNA methylation, histone composition, post-translational histone modifications, and nucleosome occupancy (Coolen et al. 2010; Taberlay and Jones 2011; Bert et al. 2013), can lead to global changes of the cancer epigenome coupled with associated deregulation of cancer gene expression signatures. DNA methylation currently remains the most characterized epigenetic mechanism in normal and cancer biology. Seminal studies have demonstrated that while CpG island-associated promoters are commonly hypermethylated and silenced (Jones and Baylin 2007; Baylin and Jones 2011), the bulk of the genome is abnormally hypomethylated in cancer cells. Genome-wide hypomethylation occurs commonly at intergenic regions and likely contributes to genomic instability during transformation (Almeida et al. 1993; Ji et al. 1997), highlighting the critical importance of the epigenome in determining chromatin structure.

The nucleosome is the organizational unit of chromatin and can therefore be considered one of the underlying drivers of the epigenetic state and ultimately, transcriptional output. As such, it is increasingly important to consider how nucleosomes are organized in the context of the entire epigenome. Pioneering studies that couple nuclease-treated DNA with genome-wide sequencing have already provided a wealth of information regarding global nucleosome occupancy (Yuan et al. 2005; Ioshikhes et al. 2006; Lee et al. 2007; Schones et al. 2008); notably, that nucleosomes are absent from the −1 position near transcriptional start sites of transcribed genes (Yuan et al. 2005; Ioshikhes et al. 2006; Lee et al. 2007; Lin et al. 2007; Whitehouse et al. 2007; Field et al. 2008, 2009; Schones et al. 2008; Shivaswamy et al. 2008; Valouev et al. 2008), but are surrounded by highly organized nucleosomes enriched in active histone modifications (Barski et al. 2007). We recently demonstrated that the strength of a promoter nucleosome-depleted region (NDR) is correlated with the associated level of gene expression (Kelly et al. 2012). Yet, the physical presence of the nucleosome is also important; they anchor DNA methyltransferases (Jeong et al. 2009; Sharma et al. 2011) that are necessary for DNA methylation (You et al. 2011) and/or carry repressive histone modifications that contribute to formation of inaccessible chromatin structures (Taberlay et al. 2011). Indeed, the key role of the nucleosome is gaining recognition in both normal and malignant promoter regulation (Kelly et al. 2010; Wolff et al. 2010; Andreu-Vieyra et al. 2011; Taberlay et al. 2011; You et al. 2011). To date, however, research has primarily focused on DNA methylation and loss of function of CpG island-containing promoters, which represent only a small proportion of the potential regulatory nodes in cancer.

Distal regulatory elements such as enhancers and insulators determine the transcriptional profile of a cell in addition to promoters. Our recent work has revealed that inactive, but permissive, enhancers exhibit a remarkably similar epigenetic signature to that of active enhancers, and are commonly paired with promoters that are repressed by the Polycomb repressive complex in order to facilitate cellular reprogramming by master regulatory factors (Taberlay et al. 2011). In contrast, we found that cancer cells are rendered resistant to cellular reprogramming if the cognate enhancer is occupied by nucleosomes and methylated. These data suggested that distal regulatory elements are also subject to epigenetic modification and could be a key feature of the cancer epigenome.

Here, we have integrated epigenome-wide maps of DNA methylomes and nucleosome occupancy in prostate and breast normal and cancer cells to gain a more comprehensive understanding of the relationship between NDRs and cancer-related epigenetic changes that occur at distal regulatory regions. We find that reconfiguration of NDRs at distal regulatory elements in cancer cells is associated with epigenetic deregulation and a change in DNA methylation of enhancers and insulator elements.

## Results

### Nucleosome-depleted regions (NDRs) are largely unique to individual cell types

**Figure 1.**
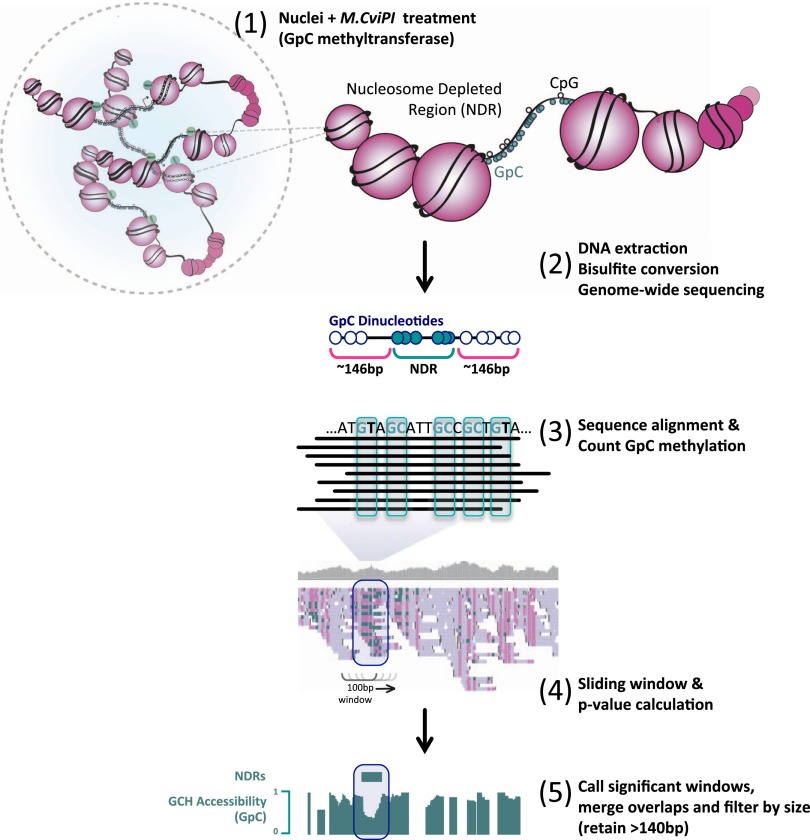
NOMe-seq reveals that patterns of nucleosome occupancy are largely unique to individual cell types. Here we provide a schematic representation of NOMe-seq methodology and the bioinformatics pipeline implemented for the detection of NDRs independent of other genomic features (e.g., TSS, DNase hypersensitivity sites) (see Methods).

We recently developed a method for high-resolution simultaneous genome-wide mapping of DNA methylation and nucleosome occupancy within individual DNA strands using nucleosome occupancy and methylome sequencing (NOMe-seq) (Kelly et al. 2012). NOMe-seq exploits the ability of M.CviPI (Xu et al. 1998) to methylate GpC sites within regions of DNA that are not occupied by nucleosomes or other tight-binding proteins (Fig. 1; Jessen et al. 2004, 2006; Hoose and Kladde 2006; Kilgore et al. 2007; Kelly et al. 2010; Andreu-Vieyra et al. 2011; Taberlay et al. 2011; You et al. 2011, 2013). Thus, NOMe-seq produces a high-resolution digital readout of endogenous DNA methylation (from CpG sites) and nucleosome occupancy or depletion (from GpC sites) within individual molecules, which removes the need for a population-based analysis to infer a relationship between DNA methylation and nucleosome occupancy. Here, we performed the first comparative analyses of NOMe-seq data from multiple cell types, including normal human mammary epithelial cells (HMEC) and a breast cancer cell line (MCF7), as well as normal prostate epithelial cells (PrEC) and a prostate cancer cell line (PC3), to define the altered patterns of DNA methylation, histone modifications, and nucleosome occupancy that occur at distal regulatory elements in two different cell line models of cancer.

First, we developed a modified version of our custom bisulfite sequencing analysis pipeline (Fig. 1; see also Methods; Statham et al. 2012) to call nucleosome-depleted regions (NDRs). These were defined as having a significant enrichment of GpC methylated sites above the background distribution across a minimum of 140 bp, independent of other genomic landmarks (e.g., transcriptional start sites), and called at increasing stringencies (range; low: −log_10_ *P*-value = 5, high: −log_10_ *P*-value = 20). We found that few NDRs were shared between the breast cell lines (HMEC and MCF7), with overlap being ∼10% regardless of the cutoff used (Supplemental Fig. S1A). The same trend was observed when we compared PrEC and PC3 prostate cells (Supplemental Fig. S1B). Based on this analysis, we selected a midrange –log_10_ *P*-value = 15 for all subsequent experiments to ensure high-confidence in NDR calls.

To further investigate the extent of unique and common NDRs, we extended our comparison to include all four cell types. Interestingly, we found that 65.84% of a total 88,578 NDRs detected were unique to only one cell type (Supplemental Fig. S1C). We tested whether this was solely due to the stringency chosen and found that <4% of NDRs are shared between all four cell types, even at the highest stringency (Supplemental Fig. S1D). These data show a plateau at –log_10_ *P*-value = 15 and that ≤20% NDRs are shared between any two cell types, regardless of the pairs. We also intersected all NDRs to show the relationship between cell types (Supplemental Fig. S1E). Taken together our data demonstrate that nucleosome occupancy at a given genomic point is largely unique to individual cell types independent of normal or cancer status.

We next asked whether the NDR calls detected by NOMe-seq overlapped accessible regions identified by DNase-seq and FAIRE-seq, which are commonly used techniques to detect the presence of accessible chromatin. A comparison of these assays revealed that NOMe-seq, DNase-seq, and FAIRE-seq each detect only a subset of accessible regions (Supplemental Fig. S2A). Notably, these disparities may be attributed to each technique having a different preference to GpC density (Supplemental Fig. S2B–D). DNase-seq has a preference for GC-rich regions (Supplemental Fig. S2B), whereas FAIRE-seq interrogates GC-depleted regions (Supplemental Fig. S2C). In contrast, NOMe-seq spans both GC-depleted and GC-rich regions (Supplemental Fig. S2D).

### NDRs are enriched at enhancers in normal epithelial cells and change to predominantly overlap CTCF in cancer cells

To determine if NDRs were differentially located at functional genomic loci between normal and cancer cells, we needed to consider NDRs in the context of epigenome maps extending beyond DNA methylation and nucleosome occupancy using NOMe-seq. We therefore performed ChIP-seq to generate signatures of key histone modifications (H3K4me1, H3K27ac, H3K4me3, H3K27me3) and regulatory factors (CTCF, RNA Pol II) and then applied the multivariate hidden Markov model, ChromHMM (Ernst and Kellis 2010, 2012; Ernst et al. 2011), to annotate the epigenomes of each normal and cancer cell type into nine distinct chromatin states (heterochromatin, repressed, transcribed, enhancers, enhancers + CTCF, CTCF, promoters + CTCF, promoters and promoter_poised) (Supplemental Fig. S3A). Each chromatin state was enriched with the expected complement of histone modifications (Supplemental Fig. S3A). Additionally, we confirmed that RNA-seq profiles surrounding each of these ChromHMM states were as expected (e.g., HMEC and MCF7) (Supplemental Fig. S3B).

**Figure 2.**
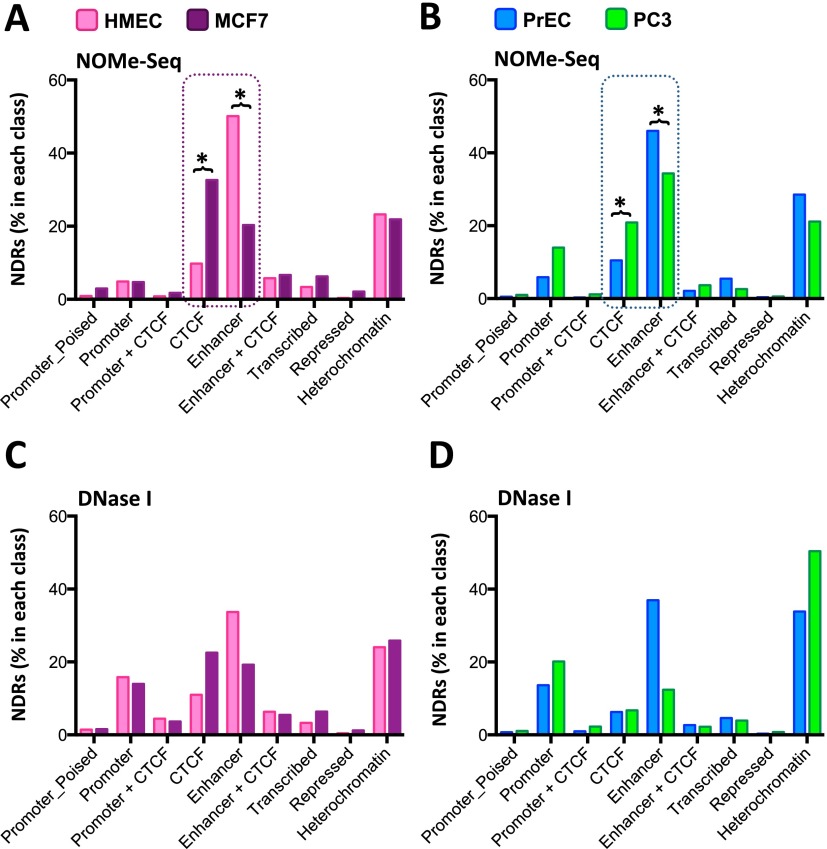
NDRs are enriched at enhancers in normal epithelial cells and change to predominantly overlap CTCF in cancer cells. (*A*,*B*) ChIP assays were performed with antibodies detecting RNA Pol II-P, H3K4me3, H3K27ac, H3K4me1, and H3K27me3 on chromatin from HMEC, MCF7, PrEC, and PC3 cells. ChIP-seq data for CTCF was generated (PC3) or obtained from ENCODE. ChIP-seq chromatin states were classified using the ChromHMM hidden Markov model (Ernst and Kellis 2012). Data are presented as percentage of NDRs detected by NOMe-seq in each of nine chromatin states (heterochromatin, repressed, transcribed, enhancer, enhancer + CTCF, CTCF, promoter + CTCF, promoter, promoter_poised) (*y*-axis) for HMEC (pink bars), MCF7 (purple bars), PrEC (blue bars), and PC3 (green bars). (*C*,*D*) As for *A* and *B* using DNase I hypersensitivity data as the measure of accessibility (percentage of NDRs in each class).

We then intersected our NOMe-seq data sets with each of the ChromHMM states and asked where NDRs were most dominant in the genome (Fig. 2). In HMEC, the majority of NDRs were found to overlap enhancers (50.12%) (Fig. 2A). In contrast, only 20.34% of NDRs overlap an enhancer in MCF7 cancer cells. Instead, most NDRs cover CTCF sites (32.66%) in MCF7 cells (Fig. 2A) compared to 9.77% that overlap CTCF sites in HMEC cells (Fig. 2A). However, the proportion of NDRs overlapping most other regulatory regions does not change between HMEC and MCF7 (Fig. 2A). Importantly, the same trend is observed in the prostate cells, where NDR locations localize more commonly to enhancers in PrEC compared to CTCF occupied regions in PC3 (Fig. 2B). We also performed the same genomic analysis, but using DNase I hypersensitive sites as the measure of accessibility (Fig. 2C,D). We again observed fewer DNase I hypersensitive sites overlapping enhancers (Fig. 2C,D) and a greater number of DNase I sites overlapping CTCF sites in cancer cells (Fig. 2C,D), supporting the NOMe-seq results (Fig. 2A,B). Our data suggest that although NDRs are mostly unique to each cell type (Supplemental Fig. S1), the genomic locations of NDRs are commonly shared between normal cells, which are predominantly at enhancers, whereas in cancer cells, NDRs are located primarily at CTCF insulator elements.

### Epigenetic silencing of regulatory elements extends to include both enhancers and insulators in cancer cells

**Figure 3.**
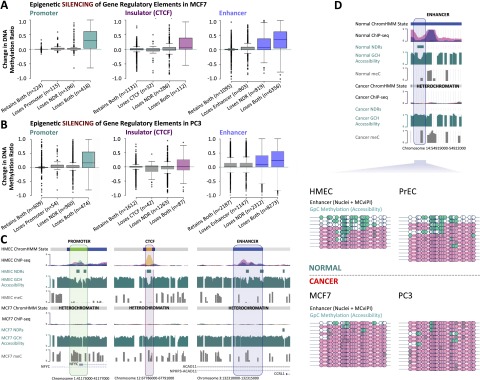
Epigenetic silencing of enhancers and insulators, in addition to promoters, in cancer cells. (*A*) Promoters (broadly, nucleosome-depleted, and H3K4me3 enriched) were defined in HMEC cells. These exact genomic regions were subject to ChromHMM classification in MCF7 cells and the extent and type of epigenetic silencing (nucleosome acquisition, loss of active epigenetic marks, and gain of DNA methylation) was determined at promoters (*left* panel, teal). Similarly, insulators (broadly, nucleosome-depleted, and CTCF; *center* panel, purple) and enhancers (broadly, nucleosome-depleted, and H3K4me1 enriched; *right* panel, blue) were defined in HMEC cells. These exact genomic regions were subject to ChromHMM classification in MCF7 cells, and the extent and type of epigenetic silencing was determined at insulators and enhancers. (*B*) As in *A*, for PrEC and PC3. (*C*) Screenshots showing epigenetic reprogramming at a promoter (*left* panel), a CTCF site (*middle* panel), and an enhancer (*right* panel). For ChIP-seq tracks: (orange) CTCF; (pink) H3K27ac; (purple) H3K4me1; (green) H3K4me3; and (red) H3K27me3. (*D*) Validation of enhancer epigenetic silencing. Representative colors for ChIP-seq tracks are as shown in *C*. All four cell types were treated with M.CviPI GpC methyltransferase and subjected to bisulfite conversion and cloning. Horizontal lines represent individual enhancers. Circles represent GpC dinucleotides: (white) unmethylated and inaccessible to M.CviPI; (teal) methylated and accessible to M.CviPI. Pink bars represent sites associated with nucleosomes. Regions accessible to M.CviPI (teal) indicate NDRs. Diagram is drawn to scale.

Our previous work revealed that the cognate enhancer of *MYOD1* is devoid of H3K4me1 and occluded by nucleosomes in cancer cells (Taberlay et al. 2011). This prompted us to ask whether abnormal changes in nucleosome occupancy, histone marks, and DNA methylation at distal regulatory elements occur on a global scale and could be considered a general characteristic of cancer cells. To address this question, we selected the promoters, CTCF sites, and enhancers that were defined in normal cells (ChromHMM; HMEC and PrEC) and then characterized the epigenetic changes that occur at these *precise* genomic regions in cancer cells (MCF7 and PC3). For example, enhancers defined in normal cells were separated into four categories based on their chromatin state in cancer cells: (1) “retains both” (NDR maintained and enhancer marks maintained in cancer cells); (2) “loses enhancer” (NDR maintained, enhancer marks lost in cancer cells); (3) “loses NDR” (NDR lost, enhancer marks maintained in cancer cells); and (4) “loses both” (NDR lost, enhancer marks lost in cancer cells) (Fig. 3A,B). The same categorization was also performed for promoters and CTCF sites. For each of these categories, we then assessed changes in DNA methylation to establish the spectrum of epigenetic alterations that can occur in cancer. At promoters, repressive epigenetic changes involved the concomitant loss of the H3K4me3 modification, loss of the NDR (that is, the acquisition of a nucleosome), and increased DNA methylation (Fig. 3A, left panel). For example, a promoter overlapping *NFYC* exhibits a NDR and a complement of active histone marks in HMEC cells but displays features of heterochromatin in MCF7 cells (Fig. 3C). A similar pattern was observed at CTCF sites, whereby epigenetic changes involved the concomitant loss of CTCF binding, acquisition of a nucleosome, and increased DNA methylation (Fig. 3A, middle panel). Figure 3C (middle panel) also shows an example of an intergenic insulator located on chromosome 12, where there is a loss of CTCF binding, a gain of a nucleosome, and gain of DNA methylation in MCF7 cells relative to HMEC cells. These data suggest that epigenetic silencing requires loss of the NDR in concert with removal of H3K4me3 or CTCF from promoters and insulators in cancer cells.

In contrast, we found that enhancer epigenetic silencing can be distinct from both promoters and CTCF sites (Fig. 3A, right panel). At enhancers, increased DNA methylation was observed in the presence of H3K4me1 but was associated with the gain of a nucleosome (“loses NDR”) (Fig. 3A, right panel). Therefore, increased DNA methylation at enhancers does not require removal of the H3K4me1 mark. The same analysis in prostate cells demonstrated that these observations were not limited to breast cells (Fig. 3B, right panel). Indeed, as we described in breast cancer cells, promoters and CTCF sites shared common features of epigenetic change, whereas enhancers displayed different behavior in prostate cancer cells (Fig. 3B). Together, our data infer that enhancer epigenetic silencing, as exemplified by increased DNA methylation, is unique from promoters and insulators since the process does not necessarily require the removal of the defining mark (H3K4me1), suggesting that loss of the NDR, alone, can predict increased DNA methylation events at enhancers in cancer cells.

We sought to validate the genome-wide findings and used the NOMe assay (Kelly et al. 2010; Andreu-Vieyra et al. 2011; Taberlay et al. 2011; You et al. 2011, 2013) to assess nucleosome occupancy changes at a distal regulatory element that were classified as an enhancer in normal cells but marked as heterochromatin in cancer cells (Fig. 3D, upper panel). We interrogated nucleosome occupancy within individual enhancer modules in the two normal and two cancer cell types. As observed in the genome-wide data, a clear NDR is evident across the enhancer in both HMEC and PrEC cells (Fig. 3D, lower panel). In cancer cells, all individual enhancer modules were occupied by nucleosomes (MCF7 and PC3), confirming that this region exhibits a closed chromatin structure, consistent with the “heterochromatin” ChromHMM state detected in the cancer cells. Together, these data support our finding that fewer NDRs overlap enhancers in cancer cells and is also consistent with our observation that a greater number of CTCF sites overlap a NDR in cancer cells (Fig. 2).

### Epigenetic activation can encompass distal gene regulatory elements

**Figure 4.**
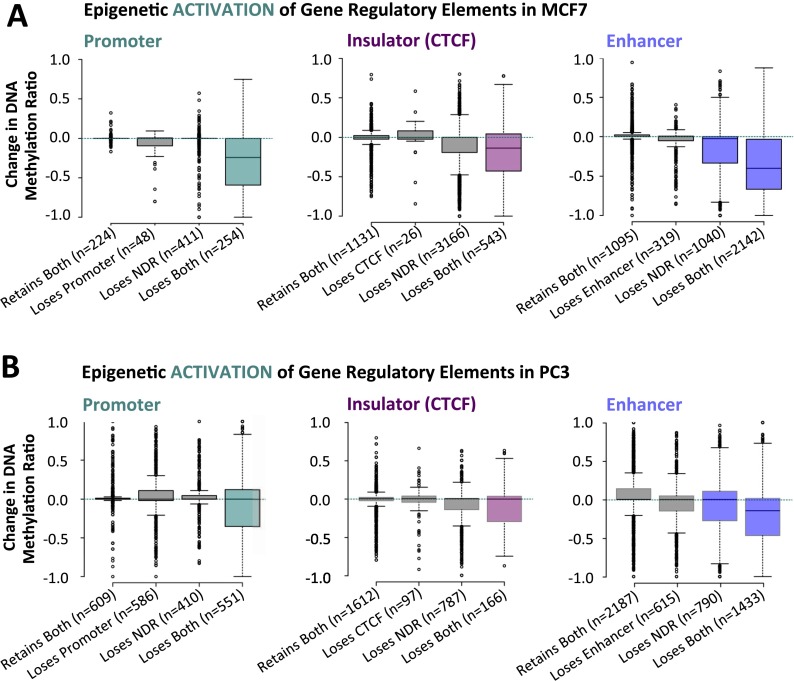
Epigenetic activation of enhancers and insulators, in addition to promoters, in cancer cells. (*A*) Promoters (broadly, nucleosome-depleted, and H3K4me3 enriched) were defined in MCF7 cells. These exact genomic regions were subject to ChromHMM classification in HMEC cells, and the extent and type of epigenetic activation (nucleosome loss, acquisition of active epigenetic marks, and reduced DNA methylation) was determined at promoters (*left* panel, teal). Similarly, for insulators (broadly, nucleosome-depleted, and CTCF; *center* panel, purple) and enhancers (broadly, nucleosome-depleted, and H3K4me1 enriched; *right* panel, blue). (*B*) As in *A*, for PrEC and PC3 cells.

We then sought to determine whether a subset of promoters, CTCF sites, and enhancers could conversely acquire an abnormally active epigenetic signature. We identified a subset of regulatory elements that either gained H3K4me3 (at promoters), CTCF binding (at insulators), or H3K4me1 (at enhancers). Indeed, we found that promoters, CTCF sites, and enhancers could all exhibit characteristics of epigenetic activation in MCF7 breast cancer cells (Fig. 4A) and in PC3 prostate cancer cells (Fig. 4B). Interestingly, we noticed that fewer regulatory elements aberrantly acquire an active epigenetic signature: For example, 2142 enhancers gain an active signature (reduced DNA methylation, nucleosome loss [NDR formed], and increased H3K4me1) (Fig. 4A), whereas 6356 enhancers become silenced (increased DNA methylation, nucleosome gain [NDR lost], and removal of H3K4me1) (Fig. 3A) in MCF7 compared to HMEC. This trend is consistent with previous findings (Akhtar-Zaidi et al. 2012) and suggests that it is more difficult to activate a silenced regulatory element than it is to silence an already active or poised regulatory element.

### NOMe-seq reveals that nucleosomes are phased throughout the genome, even at weak or absent nucleosome-depleted regions (NDRs)

**Figure 5.**
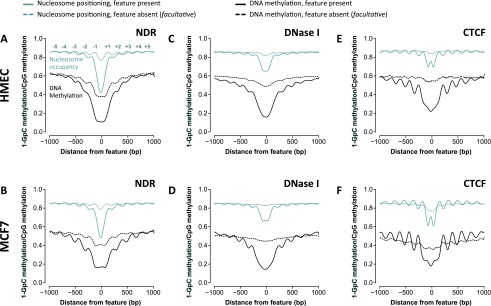
NOMe-seq reveals that nucleosomes are organized throughout the genome, at nucleosome-depleted regions (NDRs) and *facultative*NDRs (*f*NDRs). (*A–F*) Each genomic feature (NDR, DNase hypersensitivity, or CTCF binding site) was called as “present” (solid lines) or “absent” (broken lines) in HMEC (*upper* panels) or MCF7 (*lower* panels). Nucleosome occupancy and endogenous methylation (CpG) were mapped ±1000 bp from the center (“0”) of each NDR, DNase I hypersensitive site, or CTCF site. (*A,B*) NOMe-seq demonstrates that nucleosomes are present on either side of a NDR (solid teal lines) and across the genome, even at less pronounced or absent NDRs (broken teal line). DNA methylation is phased alongside nucleosomes (black lines) regardless of nucleosome occupancy; however, DNA methylation is typically low (∼20%) within NDRs (solid black line). (*C*,*D*). DNase I hypersensitive sites are characterized by organized nucleosomes (solid teal lines), whereas DNA methylation (solid black lines) is low at the center, then phased, and then increases with distance from the center of the DNase I site (solid black line) similar to patterns observed around NDRs. (*E*,*F*). CTCF is associated with nucleosome patterns (solid teal lines) and phased peaks of DNA methylation (solid black lines) between the nucleosomes. In the absence of CTCF, nucleosomes are not phased (broken teal lines), and a continuous high level of DNA methylation is observed (∼60%).

We next asked whether an underlying feature of the genome could determine the precise location of an NDR since our data point to nucleosome occupancy as a cell type-specific feature. We took advantage of the single-molecule readout of NOMe-seq to determine whether global nucleosome occupancy at a given genomic region is random or organized. We examined nucleosome phasing ±1 kb from (1) all NDRs in each cell type; and (2) all other regions of the genome that could potentially exhibit a NDR (*facultative*; *f*NDR) as defined in at least one of the four NOMe-seq data sets but not in the specific cell type examined. At NDRs, we observed phasing of at least four nucleosomes flanking the NDRs in HMEC and MCF7 cells. Notably, the nucleosome phasing matched an apparent methylation phasing with the peaks of DNA methylation occurring between the phased nucleosomes and low levels of DNA methylation at the NDR (Fig. 5A,B). Hypomethylation at NDRs and similar exquisite phasing of flanking nucleosomes and methylation patterns were also observed in the PrEC and PC3 NOMe-seq data sets (Supplemental Fig. S4A,B). This is consistent with the finding that unmethylated sequences are refractory to stable nucleosome formation (Collings et al. 2013).

Next, we mapped nucleosome phasing ±1 kb from the center of *f*NDRs (i.e., nucleosome occupied in the cell type examined but depleted in one of the other cell types). In HMEC, the precise phasing of the nucleosomes at *f*NDRs was striking and unexpectedly consistent with the nucleosome organization observed at NDRs (cf. broken teal line to solid teal line, Fig. 5A). The phasing at *f*NDRs was again pronounced in MCF7 (Fig. 5B), PrEC (Supplemental Fig. S4A), and PC3 (Supplemental Fig. S4B), confirming that this pattern was not unique to HMEC cells. To validate this unexpected finding, we next performed the same analysis using a –log_10_ *P*-value = 3 to more definitively separate NDRs and *f*NDRs. At this threshold, weak NDRs are removed but phasing is still observed in the “absent” bin (Supplemental Fig. S4G–J). Irrespective of threshold, nucleosomes flanking the *f*NDRs were also accompanied by a pattern of phased DNA methylation (Fig. 5A,B; Supplemental Fig. S4A,B).

### Nucleosomes are not phased when DNase I hypersensitive sites are absent or CTCF is not bound

DNase I hypersensitivity is a measure of accessible DNA that infers nucleosome depletion (Hogan et al. 2006; Giresi et al. 2007; Kim et al. 2007) or lack of transcription factor binding (Song et al. 2011). Using a similar approach as for NDRs (Fig. 5A,B), we analyzed nucleosome occupancy ±1 kb from (1) all DNase I hypersensitivity sites in each cell type; and (2) all other regions of the genome that have the potential to be DNase I hypersensitive (*facultative*; *f*DNase I) in at least one of the four cell types. We observed that nucleosomes were depleted from DNase I hypersensitive sites (Fig. 5C,D; Supplemental Fig. S4C,D) and DNA methylation was low (Fig. 5C,D; Supplemental Fig. S4C,D), as expected. Nucleosome phasing is evident surrounding DNase I sites in all four data sets but diminished at *f*DNase I sites (Fig. 5C,D; Supplemental Fig. S4C,D). Similarly, DNA methylation phasing is observed at DNase I and absent from *f*DNase I sites (Fig. 5C,D; Supplemental Fig. S4C,D). This is in contrast to DNA methylation phasing observed at both NDRs and *f*NDRs.

Nucleosomes are organized surrounding CTCF sites (Kelly et al. 2012; Wang et al. 2012b). Therefore, using a similar approach as for NDRs (Fig. 5A,B) and DNase I (Fig. 5C,D), we examined nucleosome phasing ±1 kb from all CTCF-occupied regions in each cell type as established by ChIP-seq. We found that CTCF binding sites are unmethylated, nucleosome-depleted, and interestingly, display a small region of inaccessibility at the peak center, corresponding to a footprint of the bound CTCF complex (Fig. 5E,F; Supplemental Fig. S4E,F). As expected, nucleosomes and DNA methylation were strongly phased surrounding CTCF binding sites. However, a further ∼5% dip in DNA methylation was observed at the peak of CTCF binding.

Whether nucleosome distribution is perturbed in the absence of CTCF binding has not been addressed. Therefore, we also examined all other regions of the genome that have the potential to be occupied by CTCF (*facultative*; *f*CTCF) in at least one of the four cell types. At *f*CTCF sites, where CTCF is not bound, nucleosome phasing is not evident (Fig. 5E,F; Supplemental Fig. S4E,F). Consistent with a lack of organized nucleosomes (Fig. 5E,F; Supplemental Fig. S4E,F), *f*CTCF sites do not exhibit DNA methylation phasing (Fig. 5E,F; Supplemental Fig. S4E,F). Global DNA hypomethylation was evident in both MCF7 and PC3 cancer cells surrounding CTCF and *f*CTCF sites as well as the NDRs and DNase I sites.

### Transcriptional regulator binding sites are nucleosome-depleted, hypomethylated, and associated with precise nucleosome phasing

**Figure 6.**
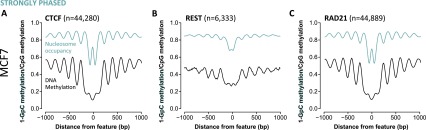
CTCF, RAD21, and REST are associated with patterns of nucleosome organization. (*A–C*) Nucleosome occupancy (teal lines) and endogenous methylation (CpG; black lines) were mapped ±1000 bp from the center (“0”) of each transcription factor binding site (CTCF, REST, and RAD21) and were determined to be “strongly phased” in MCF7 cells.

In addition to CTCF, nucleosome phasing has also been detected at other transcriptional regulator binding sites (Wang et al. 2012b), raising the possibility that nucleosome phasing surrounding regulatory factors may be widespread. To determine the extent of nucleosome organization, we mapped nucleosome occupancy and DNA methylation adjacent to the peak center of 20 transcriptional regulators from publicly available MCF7 data sets (Supplemental Table 1). We examined nucleosome phasing ±1 kb from each transcriptional regulator binding site and observed marked nucleosome depletion in all data sets (Fig. 6A–C; Supplemental Fig. S5A,B). DNA methylation was low (typically ∼5%–10%) at the center of transcriptional regulator binding, despite many of these factors not containing CpG sites in their consensus sequence. Of the 20 transcriptional regulators we examined, only three were associated with strong nucleosome phasing: CTCF, REST, and RAD21 (Fig. 6A–C); whereas we observed the phasing of at least five nucleosomes flanking the NDR. This was accompanied by DNA methylation phasing, whose peak corresponded with the trough in the nucleosome array (consistent with linker regions) (Fig. 6A–C). Interestingly, small patches of inaccessibility corresponding to footprints created by CTCF, REST, or RAD21 binding and the associated dip in DNA methylation were only observed in the presence of strongly phased nucleosomes (cf. Fig. 6A–F and Supplemental Fig. S5A,B). In contrast ELF1, FOSL2, GABP, JUND, HDAC2, and MAX corresponded with weak nucleosome phasing and almost indiscernible phasing patterns in DNA methylation (Supplemental Fig. S5A), whereas nucleosome phasing was not detected at the remainder of the transcriptional regulator binding sites (Supplemental Fig. S5B). Taken together, these data suggest that only certain regulatory factors play key roles in establishing nucleosome and DNA methylation phasing patterns.

## Discussion

**Figure 7.**
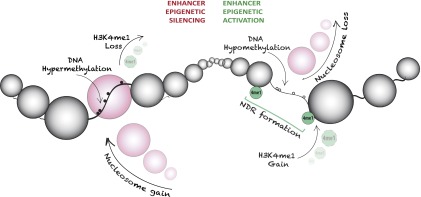
Model depicting mechanisms by which the distal regulatory architecture provides an additional layer of epigenetic plasticity in cancer. Enhancers preferentially undergo epigenetic silencing, which is exemplified by collapse of a NDR (nucleosome gain), DNA hypermethylation, and loss of the H3K4me1 mark. Epigenetic switching can also encompass aberrant epigenetic activation of enhancers. Here, an atypical NDR is formed due to nucleosome loss, and the enhancer becomes marked by H3K4me1, which is accompanied by DNA hypomethylation events in the cancer cells. Our data indicate that many more enhancers are abnormally silenced than activated, consistent with the hypothesis that it is more difficult to activate an already silenced regulatory element than it is to silence an already active or poised regulatory element. These findings also support a permanently silenced state in cancer. Thus, our data demonstrate that the global epigenetic landscape is dynamically altered at key regulatory regions outside of promoters. (NDR) Nucleosome-depleted region; (small white circle) unmethylated CpG site; (small black circle) methylated CpG site; (large circle) nucleosome; (4me1 [green]) H3K4me1.

The critical events that underlie epigenetic aberrations at promoters have been largely deciphered (for review, see Jones and Baylin 2007; Baylin and Jones 2011; Taberlay and Jones 2011); yet, epigenetic alterations encompassing nucleosome occupancy, histone marks, and DNA methylation at distal regulatory regions have not been similarly detailed. We utilized comprehensive epigenome-wide maps of DNA methylomes and nucleosome occupancy (NOMe-seq) in cell line models of prostate and breast cancer to gain an understanding of nucleosome distribution in each cell type and define a spectrum of epigenetic changes that occurs at distal regulatory regions. Here, we show that NDRs and key TFs play an important role in phasing nucleosomes and establishing flanking DNA methylation patterns. Moreover, we find that in cancer cells, “decommissioning” of NDRs and TFs at distal regulatory elements is associated with DNA hypermethylation of enhancers and insulator elements and gain of repressive epigenetic marks (Fig. 7). In addition we show that loss of nucleosomes at distal regulatory elements is associated with demethylation and gain of active epigenetic marks (Fig. 7). Our study emphasizes the importance of understanding epigenetic control in the context of the entire epigenome to determine how genomic circuits typified by distal regulator elements may be disrupted during malignancy.

Foremost, and irrespective of normal or cancer status, NOMe-seq methodology allowed us to observe that the phasing of nucleosomes occurs directly with phasing of DNA methylation, which is not possible with other assays that determine accessibility, such as DNase-seq or FAIRE-seq. We observed that phasing occurs in all cell types, although it remains unclear what is dictating the precise organization that is evident at NDRs and even at absent NDRs (*f*NDRs). The first possibility is a sequence-based propensity for DNA to be associated with nucleosome occupancy (Bernstein et al. 2004; Segal et al. 2006; Kaplan et al. 2009; Kelly and Jones 2011; Hughes et al. 2012; Struhl and Segal 2013) at these regulatory elements, supporting a model whereby nucleosome organization may be more conserved than currently anticipated. Our data also point to a second scenario, whereby select regulatory factors are also critical for organizing nucleosomes and subsequently, DNA methylation patterns. All the transcription factor binding sites that we examined displayed a NDR that is conspicuously unmethylated. Although the majority of transcription factors showed little or no evidence of nucleosome or peripheral DNA methylation phasing, nucleosome and DNA methylation phasing is highly pronounced around CTCF, REST, and RAD21 binding sites. These sites display a patch of inaccessibility at the binding midpoint, consistent with a transcription factor footprint observed in previous reports using DNase-seq (Neph et al. 2012; Wang et al. 2012b). That DNA methylation is highly phased in concert with organized nucleosomes surrounding these transcription factor binding sites is consistent with the observation that linker regions are more accessible to DNA methyltransferases (DMTases) (Felle et al. 2011), as we have observed (Kelly et al. 2012) and shown to be due to “seeding” methylation that occurs preferentially between nucleosomes at linker regions and then spreads to nucleosomes (Hinshelwood et al. 2009). Interestingly, our data show that the pattern of DNA methylation phasing is highly consistent with the strength of the nucleosome phasing, further highlighting the strong interaction between DMTases and nucleosomes. However, it should be noted that many transcription factor recognition sequences do not contain CpG sites and therefore, DNA methylation may not be directly influencing their binding profiles, as has been reported for MYC (Prendergast and Ziff 1991; Prendergast et al. 1991). It will be important to consider in future studies whether the strength, stability, or physical bulk of the transcription factor binding complexes and or the genomic sequence context can explain why only some regulatory factors are associated with highly organized nucleosomes flanking NDRs.

Nucleosome-depleted enhancers are critical for the activity of master regulators that determine cellular phenotype (Taberlay and Jones 2011). Here we show that very few NDR locations are shared between cell types, consistent with observations that the majority of enhancers are cell type-specific (Heintzman and Ren 2007; Visel et al. 2007; Heintzman et al. 2009). Categorization of the epigenome into ChromHMM states showed that NDRs are predisposed to overlap enhancers in normal cells; however, we found that NDRs preferentially overlapped CTCF sites in cancer cells. The question remains as to the underlying biological impact of this substantial reorganization of NDRs in cancer cells. It has been proposed that architectural proteins such as CTCF may be associated with widespread changes to the cancer methylome in addition to alterations in the physical packaging of chromatin (Berman et al. 2012) and perhaps phasing. Our results support this hypothesis, because we can only detect phasing when CTCF occupies its cognate binding site, whereas both nucleosome phasing and DNA methylation patterns are disrupted when CTCF is not bound. This suggests that CTCF, and most likely RAD21 and other factors such as REST, are driving the arrangement of nucleosomes and perhaps dictating DNA methylation patterns, offering a potential explanation for the substantial reorganization of NDRs to primarily overlap CTCF in the cancer cells.

This may be particularly important in the context of cancer in which global hypomethylation occurs concomitant with aberrant, but targeted, epigenetic silencing of discrete genomic regions such as promoters (Taberlay and Jones 2011). Indeed, it has long been accepted that epigenetic switching is a hallmark of promoters in cancer cells (e.g., Gal-Yam et al. 2008). We now show that epigenetic silencing of distal regulatory elements is achieved through increased DNA methylation, altered nucleosome occupancy, and loss of characteristic signatures (such as H3K4me1 or CTCF that largely define enhancers or insulators, respectively). Although current technologies limit our ability to dissect a specific order of events, we have previously demonstrated that nucleosomes first occupy enhancers, prior to DNA methylation events (You et al. 2011). Since enhancers are typically CpG-poor (Andreu-Vieyra et al. 2011; Stadler et al. 2011; Jones 2012), our data reveal that aberrant epigenetic silencing or activation of these distal regulatory elements is not directly governed by CpG density. Our analysis shows that although distal enhancers can be aberrantly silenced or activated in cancer (Fig. 7), the data indicate that the process may be distinct from promoters and insulators. Foremost, our data suggest that the enhancer-associated H3K4me1 mark may not be mutually exclusive with DNA methylation, unlike H3K4me3, and that a change in nucleosome occupancy (gain or loss) may be sufficient to trigger epigenetic changes at enhancers.

The study of two cancer cell line models allowed a detailed and methodical investigation, showing reconfiguration of nucleosome-depleted regions at distal regulatory elements accompanies epigenetic changes that occur at enhancers and insulators. It is interesting to consider that the molecular changes we have observed in this analysis of breast and prostate cancer cells may explain some of the gross alterations that occur in tumors and are visible to pathologists. Taken together, our findings suggest that disruption of the local nuclear architecture at enhancers and insulators offers a novel role for epigenetic reprogramming distant from promoters in cancer cells.

## Methods

### Cell culture

Normal human prostate epithelial cells (PrEC), a prostate cancer cell line (PC3), and a breast cancer cell line (MCF7) were obtained from the American Type Culture Collection (ATCC). Normal human mammary epithelial cells (HMEC) were obtained from Invitrogen. All cell lines were cultured under recommended conditions at 37°C and 5% CO_2_.

### Nucleosome occupancy and methylation assay (NOMe Assay)

NOMe assays were conducted as described previously (Taberlay et al. 2011). Briefly, cells were trypsinized and centrifuged for 3 mins at 500*g*, then washed in ice-cold PBS and resuspended in 1 mL ice-cold Nuclei Buffer (10 mM Tris, pH 7.4, 10 mM NaCl, 3 mM MgCl_2_, 0.1 mM EDTA, and 0.5% NP-40, plus protease inhibitors) per 5 × 10^6^ cells and incubated on ice for 5 min. Nuclei were recovered by centrifugation at 900*g* for 3 min and washed in Nuclei Wash Buffer (10 mM Tris, pH 7.4, 10 mM NaCl, 3 mM MgCl_2_, and 0.1 mM EDTA containing protease inhibitors). Freshly prepared nuclei (2 × 10^5^ cells) were resuspended in 1× M.CviPI reaction buffer (NEB), then treated with 150 units of M.CviPI (NEB; 50,000 units/mL) in 15 μL 10× reaction buffer, 45 μL 1M sucrose, and 0.75 μL SAM in a volume of 150 μL. Reactions were quenched by the addition of an equal volume of Stop Solution (20 nM Tris-HCl [pH 7.9], 600 mM NaCl, 1% SDS, 10 mM EDTA, 400 μg/mL Proteinase K) and incubated overnight at 55°C. DNA was purified by phenol/chloroform extraction and ethanol precipitation. Bisulfite conversion was performed using the EpiTect Bisulfite Kit (Qiagen). For analyses of individual genomic regions of interest, PCR amplicons were cloned using the TOPO TA Kit (Invitrogen) and then sequenced. Oligonucleotides used for enhancer epigenetic silencing were:

Enh14For 5′-TATTTTTATTATTAGGAATATTTGTAATTTTTTTAAG-3′
Enh14Rev 5′-AACCTCTACTTTATTTAATAATTTCTTCA-3′

### Nucleosome occupancy and DNA methylation sequencing (NOMe-seq)

Libraries for genome-wide NOMe-seq analyses were prepared using the established protocols of the USC Epigenome Center. Briefly, genomic DNA (2 μg) was sonicated using a Covaris instrument to an average molecular weight of 150 bp. Achievement of the desired size range was verified by Bioanalyzer analysis (Agilent Technologies). Fragmented DNA was repaired to generate blunt ends using the END-It kit (Epicentre Biotechnologies) according to the manufacturer’s instructions. Following incubation, the treated DNA was purified using AMPure XP beads (Agencourt). Magnetic beads were used for all nucleic acid purifications in subsequent steps. Following end repair, A-tailing was performed using the dA-tailing module according to the manufacturer’s instructions (New England Biolabs). Adapters with a 3′ “T” overhang were then ligated to the end-modified DNA. Modified Illumina paired-end (PE) adapters were used. Ligation was carried out using ultrapure, rapid T4 ligase (Enzymatics) according to the manufacturer’s instructions. The final product was then purified with magnetic beads to yield an adapter-ligation mix. Prior to bisulfite conversion, bacteriophage lambda DNA that had been through the same library preparation protocol described above to generate adapter-ligation mixes was combined with the genomic sample adapter ligation mix at 0.5% w/w. Adapter-ligation mixes were then bisulfite converted using the Zymo DNA Methylation Gold kit (Zymo Research) according to the manufacturer’s recommendations. The final modified product was purified by magnetic beads and eluted in a final volume of 20 μL. Amplification of one-half the adapter-ligated library was performed using Kapa HiFi-U Ready Mix under the following conditions: 98°C for 2 min, followed by four cycles of 98°C for 30 s, then 65°C for 15 s and 72°C for 1 min with a final extension for 10 min in 50 μL total reaction volume. The final library product was examined on the Agilent Bioanalyzer then quantified using the Kapa Biosystems Library Quantification kit according to the manufacturer’s instructions. Optimal concentrations to get the right cluster density were determined empirically. Resulting libraries were plated using the Illumina cBot and run on the Illumina HiSeq 2000 platform configured for 100-bp paired-end reads according to the manufacturer’s instructions. Read alignment was performed by the USC Epigenome Center (University of Southern California, Los Angeles) and analysis was performed using a modification of our existing bisulfite-sequencing pipeline (Statham et al. 2012) and custom scripts. QC data are provided in Supplemental Table 2. As described previously (Kelly et al. 2012), it is necessary to exclude GCG and CCG dinucleotides prior to analyses. This is particularly important since cytosine methylation cannot be designated endogenous (CpG) or enzymatic (GpC; from M.CviPI treatment) within GCG sites, while CCG can be the site of spurious methyltransferase activity (Kelly et al. 2012). GCGs equate to <0.24% of the genome and make up only 5.6% of all GpC sites, leaving 94.4% of GpC sites intact for interrogation. On average, there is one GpC dinucleotide every 20 bp (average, five GpC sites per 100 bp). Specifically, 78% of the genome has from one to six GpC sites per 100 bp (Supplemental Fig. S6A) and detected NDRs (−log_10_ *P*-value ≥15 in any of the four samples) peak at six to 13 GpC sites (Supplemental Fig. S6B). In addition, ∼93% of GCGs contain a GCH within 20 bp (∼46% being within 5 bp) (Kelly et al. 2012). Some depletion of GC-rich regions occurs in our data (Supplemental Fig. S6C), as expected (Ross et al. 2013); however, GpC-rich regions (e.g., >10 GpC sites per 100 bp bin) account for only 2.06% of the human genome (Supplemental Fig. S6A) and therefore have a minimal effect on our analysis.

### Detection of nucleosome-depleted regions (NDRs)

The number of C and T nucleotides sequenced were counted at each GCH site in the genome, and sites with less than 5× coverage in all samples were discarded from further analysis. C and T counts were summed in 100-bp windows at 20-bp spacing and tested for difference to the genome background using the χ^2^ test. Significant windows were scored at increasing *P*-value cutoffs (from −5 log_10_ to −20 log_10_), overlapped and only retained as NDRs if they were a minimum of 140 bp in size.

### Chromatin immunoprecipitation (ChIP)

ChIP assays were performed as previously (Oakford et al. 2010; Taberlay et al. 2011). Briefly, nuclei were purified (described above for NOMe-seq) after formaldehyde crosslinking, collected, and resuspended in SDS Lysis buffer before sonication. Antibodies (10 μg) used for ChIP experiments included H3K4me1 (#39298, Active Motif), H3K4me3 (#39160, Active Motif), H3K27me3 (#39155, Active Motif), H3K27ac (#39297, Active Motif), RNA Polymerase II (#ab817, Abcam), CTCF (#07-729), and CD8 (#sc-32812, Santa Cruz). Libraries for ChIP-seq were prepared by the USC Epigenome Center following Illumina protocols. The resulting libraries were sequenced on the Illumina HiSeq 2000 platform configured for 50-bp single-end reads. Public data were downloaded from ENCODE (Supplemental Table 1; Meyer et al. 2011; Thurman et al. 2012; Wang et al. 2012a; Gertz et al. 2013). Bowtie (Langmead et al. 2009) was used to align ChIP-seq reads to hg19 as previously described (Bert et al. 2013) allowing up to three mismatches, discarding reads mapping to multiple positions in the genome and removing clonal reads. ChromHMM (Ernst and Kellis 2012) was applied to the chromatin modification, CTCF, and RNA polymerase II-aligned reads to simultaneously partition the genome of all four cell lines into 15 chromatin states. Redundant states were then collapsed into nine distinct states and manually annotated by comparison to the published ChromHMM model for HMEC cells (Ernst et al. 2011). Transcription factor peaks were called using HOMER version 4.1 (Heinz et al. 2010) under the default parameters. DNase I peaks were called using Hotspot version 3 (John et al. 2011) with an FDR cutoff of 0.01.

## Data access

Raw and processed NOMe-seq and ChIP-seq data have been submitted to the NCBI Gene Expression Omnibus (GEO; http://www.ncbi.nlm.nih.gov/geo/) under accession number GSE57498.

## Competing interest statement

NOMe-seq has been commercialized by Active Motif. T.K.K. is now an employee of Active Motif, Inc., but was not at the time of completion of this work.
